# Narrativity and medicine: some critical reflections

**DOI:** 10.1186/s13010-019-0078-3

**Published:** 2019-07-15

**Authors:** Rolf Ahlzén

**Affiliations:** Department of Historical, Cultural and Religious Studies, Psychiatric Open Care Unit, Region of Värmland, Karlstad University, Bryggaregatan 1, S-65340 Karlstad, Sweden

**Keywords:** Narrativity, Narrative, Narrative medicine, Empathy, Clinical encounter

## Abstract

During the last three decades there has been a wave of interest in narrative and narrativity in the humanistic and the social sciences. This “narrative turn” has spilled over to medicine, where narrative medicine has gained a considerable influence.

However, there have also appeared second thoughts on the role of narratives in our lives, as well as on what narratives may mean in relation to clinical medicine.

This article presents some influential voices in this debate and scrutinizes the assumptions of narrative medicine in the light of these. It is concluded that there are sound reasons to tread this path with some caution and avoid the too far reaching ambitions on behalf of narrativity in relation to clinical medicine. However, narrative medicine should still be seen as a promising attempt within the broader scope of medical humanities to emphasize the importance of human subjectivity in clinical medicine.

## Introduction

Since at least thirty years, there has been a wave of interest in the meaning and role of narrative in human lives. Philosophers, literary theorists, sociologists and historians have increasingly claimed that narrative, and related notions like narrativity and narrative competence, are essential to our understanding of our personal lives, of the role of ethical values and of human suffering. Given such claims, it is not difficult to understand why narrative and narrativity soon became central to some of those who were striving to reform medicine, in order to contribute to its ability to reach its ethically defined goal of reducing suffering. Hence, Rita Charon launched her narrative medicine project with far ranging reform ambitions. The aim was to increase the narrative competence of physicians mainly by being in the company of literary texts, and also by bringing them “skills” to interpret texts and patient discourse. Narrative medicine has been influential and contributed to an almost unreserved belief in the central assumptions that underpin the “narrative movement”.

However, since a decade or more, there have been increasing doubts about several of the central assumptions of this narrative wave, which I will in the following call NW. These second thoughts have focused on both claims concerning the role of narrative in the lives of persons, and also on the question whether narrative competence really gives such a privileged access to the" first person perspective" as is often assumed. If this critique can be shown to be well founded, a substantial part of the foundation for the aspirations of narrative medicine would be undermined. It is therefore of great interest to the development of medical humanities to be aware of and attempt to judge the value of the arguments on both sides.

This article has the character of an introduction into a debate which has been going on for several years in very different disciplines and areas. It must at once be made clear that there exists no universally accepted definition of the concept narrative and that several interpretations may be around at the same time, depending on field of inquiry. My intention is to scrutinize some of the assumptions that have underpinned the growing interest in narrative medicine. It is my firm impression that this debate has passed unattended by several of those engaged in understanding narrativity and medicine. I have chosen critical voices, as I believe that they raise important questions which must be dealt with in order for the role of narrative and narrativity in medicine to be properly understood.

The article starts with a short background to the wave of interest in narrativity since the 80’s. It proceeds to outline some of the central arguments of two of the most influential critics of this narrative turn, Galen Strawson and Angela Woods. There have, however, also been promising attempts to rescue the role of narrative in a more modest form, and two of these will briefly be outlined. With these debates spelled out, narrative medicine will be presented, as it has been shaped primarily by Rita Charon. Three central assumptions of narrative medicine are identified, analyzed and found to be only partially tenable. The article will finally conclude that even with substantially more modest ambitions, narrative medicine is still one of the most interesting and promising attempts to revive subjectivity as central to medical practice.

## The early narrative wave

“Talk of narrative is intensely fashionable”, Galen Strawson wrote in 2004 ([1], p., 428). By then, the concept had been on the rise for at least a couple of decades. When the interest grew in the mid-eighties, it was clearly related to post-modern/post-structuralist thought in all its varieties, which was at that time at the peak of its influence. Psychologist Jerome Bruner in 1987 published an article in *Social Research* titled “Life as Narrative”. Bruner here emphasized the constructive character of the understanding of our lives, how stories shape interpretations and in doing so, reflect “cultural conventions and language usage” ([2], p., 15). He also conjectured that: “… eventually the culturally shaped cognitive and linguistic processes that guide the self-telling of narratives achieve the power to structure perceptual experience, to organize memory, to segment and purpose-build the very ´events´ of a life” [2]. In his widely influential essay “The Narrative Construction of Reality” the ambition was even more far ranging [3]. Narrative became the basic template for organizing reality.

These are no modest ambitions for the role of narrative. In the article, Bruner neither defines the concept of narrative, nor does he distinguish it from story. It seems as if he assumes a common sense understanding of “narrative” as being almost similar to “any ordering of a sequence of events in a temporal pattern”. If so, a story, just as a narrative, may be a verbal account of the making of a cup of tea, the purchase of a new car, a clinical intervention – as well as a shorter or longer period of time in a person’s life as it is understood and retold by her. It is the latter understanding of narrative as life forming and meaning-shaping that he is most interested in and which was emphasized by philosophers like Paul Ricoeur and Charles Taylor. The latter talks of the necessity that we “grasp our lives in a narrative”, in order to see our lives as an “unfolding story” [4]. Philosopher Angela Woods recently noted how massive the spread and influence of the idea of narrative became: “The voluminous scholarship on narrative – from philosophy, psychology, narratology, anthropology, sociology, literary and cultural studies, healthcare, law, and education – has demonstrated its centrality to understanding ´the rich and messy domain of human interaction´” [5].

In the light of this remarkable rise of a concept, it is interesting to note that prominent scholars of the previous generation did not seem to have any need to employ it. Wayne Booth, just to take an example, wrote his influential *The Company We Keep: An Ethics of Fiction* in 1988 and the concept is hardly even mentioned. The same goes for Louise Rosenblatt who in *Literature as exploration,* with the first edition appearing in 1965, does not use it at all. Narrative and narrativity also play a very inconspicuous role in the writings of Stephen Toulmin and Martha Nussbaum (as for example in the latter’s *Love’s Knowledge)*.

There are two assumptions made by the advocates for a strong role of the concept of narrativity: One is straightforwardly descriptive, saying that this is actually what we all do, more or less: we create our provisional life meaning out of narratives, by which we order and evaluate the chaotic series of events that fill our daily life. This narrative, or these narratives, may shift and transform by facing new situations and new shifts in life, calling for reinterpretation. Possibly, several such forming stories may exist at the same time, potentially creating an amount of inner tension. This assumption is, at least theoretically, possible to empirically justify or falsify.

The second claim is a normative one. It does not only hold that we *do* think of ourselves and others in the template of narrative(s), but that it is essential for a good life that we do so. From this follows that a failure to create the meaningful narrative of one’s life, or certain crucial phases in one’s life, is problematic and makes life less worth living. We shall soon see that both these assumptions are strongly rejected by Galen Strawson.

It should, however, be remembered that within what I have here called NW, the Narrative Wave, there have been considerable differences concerning which is the scope of the narrative claims. Not all who take an interest in narratives would be prepared to defend the two theses that Strawson assumes as central to the NW. We will soon see that the positions on narrativity range all the way from the minimal, “weak”, which may to some seem almost trivially true, over middle range assumptions, to “strong narrativity theses”, where, as seems to be the case for Jeremy Bruner, almost all aspects of human experience are best seen as narratively structured.

It is remarkable that, in spite of its ubiquitous use, the concept of narrative and narrativity is seldom defined in a distinct way. If it only means “any temporal ordering of a sequence of events”, then of course narrative is an outflow of human temporality and hence inevitably present all the time in human experience. But often the concept is used with far greater ambitions. A narrative is then a story, an ordering process, a binding together of events and experiences in temporal and causal chains, which give meaning and coherence to human lives. Ideally, for many “strong narrativists”, such stories together form a larger narrative, a life narrative, in the light of which one’s own existence may be seen. Not surprisingly, those who propose such a role for narratives are inclined to believe that disease, at least serious disease, “breaks” these narratives and shatters a person’s meaning of life.

## The critique

Few articles on narrativity have been more influential and created more controversy than Galen Strawson’s *Against narrativity*, published in *Ratio* in 2004. Not that the narrative wave at that time was uncontested, but Strawson’s polemic may still be seen as somewhat heretic, written by a person who does not accept what was at that time a broad consensus in large parts of the social scientific and humanistic research communities. In addition, Strawson is unconventional in several respects, as he writes extensively about literary and psychological issues while being a philosopher.

So, what are the main problems with the narrative turn, according to Strawson? His answer is that it is built on false premises, which seriously distort our understanding of the role of narratives in our lives, that is: on a number of misunderstood assumptions. So why is this then the case? The answer is characteristic for Strawson’s polemic style:

I also suspect that those who are drawn to write on the subject of “narrativity” tend to have strongly Diachronic and Narrative outlooks or personalities, and generalize from their own case with that special, fabulously misplaced confidence that people feel when, considering elements of their personality that are existentially fundamental for them, they take it that they must also be fundamental for everyone else [p. 428].

There are some distinctions in what Strawson writes which must be understood correctly in order to fully understand his argument. The first one is the identification of two theses in the discourse on narrativity, mentioned above as the Psychological and the Ethical. The second distinction is between two basic kinds of personalities, two ideal types of how people perceive themselves and their lives in temporal perspective: one is the Diachronic, who views her life as a continuity, and as something “that was there in the (further) past, and will be there in the (further) future.” [p. 430] The person who is Episodic, on the contrary, does not have this sense of continuity of the self, of having been there far back in time or that he will be there in the future, *as that self*.

To make this intelligible, it is important to note a third distinction made by Strawson, the one between oneself as a human being, “as a whole”, and oneself as a “mental entity” or “self” of some sort. It is trivially true that I see myself as continuous over time as a physical being, as a person as a whole, but according to Strawson this is not at all the case with the sense of being a “self” with continuity over time. The Episodic person just does not have that experience. Strawson emphasizes that there are, of course, combinations of these ideal types, and that this may change over time, but still assumes that most of us are basically one type or the other: “I take it that the fundamentals of temporal temperament are genetically determined and that we have here to do with a ‘deep individual difference variable’, to put it in the language of experimental psychology”. He concludes that “Diachronics and Episodics are likely to misunderstand one another badly.” [p. 431].

Finally, Strawson attempts to relate the two psychological types to Narrativity. It seems obvious that Diachronics tend to be narrative. Their sense of continuity may even take the form of a narrative, a “life story”. This is not the case with the Episodics. Strawson takes himself as an example, calling himself “fairly Episodic”. “… it’s clear to me”, he writes, “that events in my remoter past did not happen to me”, and by “me” he means not the physical being GS but his self, the mental entity GS. “I have absolutely no sense of my life as a narrative with form, or indeed as a narrative without form. Absolutely none.” [p. 433].

It remains to be made clear which definition of the concept “narrative” is employed by Strawson. This is of course a crucial question, if we want to understand the relevance for narrative medicine of his analysis. Strawson deals with the question surprisingly late in his article, and he is not very precise. A narrative, he writes, attributes “…a certain sort of *developmental* and hence temporal *unity* or *coherence* to the things to which it is standardly applied – lives, parts of lives, pieces of writing.” But to be a Narrative it cannot be enough to be able to give some sort of sequential record of how one’s life has evolved. There must also be a “*form-finding* tendency”, an ability and a need to construe a pattern, to seek coherence. To this the Narrative links a “*story-telling* tendency” when attempting to comprehend one’s life and parts of one’s life. Finally, Strawson believes there is often a tendency for *revision* among Narratives. Not all revise their stories, and not always opportunistically, but some certainly do, and this also implies a great risk for the “story-tellers” [p. 441].

It is clear that Strawson’s idea of narrativity extends beyond the most commonly applied definitions of narrative. He would of course agree that an account of a series of events, like the plot of a novel or a patient’s story of her illness, may be called a narrative. His objections to the narrativity thesis are aimed at those with greater ambitions, which he captures in his two theses. There remains, however, a vagueness in his way of dealing with this and we shall soon see that this is where his critics direct their objections, and that is also what Strawson anticipates in his article.

To summarize, Galen Strawson launches an eloquent attack on a number of assumptions concerning the role of narrative in our lives. Not only does he question the assumption that all people are basically narrative, but he also doubts that those who are get any benefits from it. “… the Narrative tendency to look for story or narrative coherence in one’s life is, in general, a gross hindrance to self-understanding: to a just, general, practically real sense, implicit or explicit, of one’s nature” [p. 447].

Philosopher Angela Woods responds to both Galen Strawson and Arthur Frank in a dense chapter, “Beyond the wounded storyteller: Rethinking narrativity, illness and embodied self-experience”. Woods shares Strawson’s skepticism concerning “the narrative turn”, but in contrast to him, she is particularly interested in the role of stories in illness. She emphasizes that narrative, apart from being expected to humanize clinical medicine, also “serves the purposes of the medical humanities as an interdisciplinary inquiry” ([5], p., 114). As the concept is broad and is extremely hard to define, it may be used by scholars from very different disciplines and thus acts as a kind of unifying concept for an intensely multidisciplinary field.

Woods sets out to challenge “two dogmas of narrative”: “The first is the claim that we are narrative selves. The second, related dogma is that the best and most healthy way to respond to illness is through narrative.” [p. 114] She accepts Strawson’s critique on major points, but notes that he is strikingly uninterested in bodily experiences and reactions to bodily change, like in illness. She laconically writes that “…the centrality of our fleshy materiality will be impossible to ignore”, and in her further analysis, she not surprisingly sets Strawson’s text in relation to the widely influential work of Arthur Frank, in particular his metaphor “broken stories”, which is supposed to capture the reality of falling ill and attempting to make some meaning out of it. As we will soon develop further, Frank’s proposal that the reaction to illness takes the shape of basically three ideal types of stories has mostly been accepted without critical scrutiny. She notes that in total contrast to Strawson, Frank thinks that we are necessarily all narrative in our responses to illness and also that this is a good thing and that the capacity to create a meaningful, constructive story, a “quest story”, of what happens in illness largely decides the possibility to heal or live with an illness. Woods summarizes Frank’s position succinctly:

For Frank, differences in the origin and even nature of the injuries sustained by the body-self, or, for that matter, very real material and cultural differences between bodies, are irrelevant because self-expression through narrative is fundamentally healthy, desirable, and even necessary, “*for everyone.*” [p. 119].

The positions of Strawson and Frank are separated by a void. Woods is closer to Strawson’s position, but develops it in an interesting way. “There is no room in Frank’s account for not being Narrative or choosing not to narrate.” [p. 122] For Woods, narrative is not necessarily the privileged form for the restitution of self-experience in illness. She sympathetically quotes Swedish researcher Lars-Christian Hydén, who writes that narrative is simply “… one of several cultural forms available to us for conveying, expressing, or formulating our experience of illness and suffering” ([6], p., 64). The limits of narrative have to do with the limits of language. Non-verbal forms of understanding and of expression may be of as great or greater importance than those of verbally mediated narrative. Narrative imposes structure, coherence, and unity where there are no such things. Narrative forces patterns on what lacks pattern. Frank would say that this is exactly why narrative, if it finds the right form, heals. Woods and Strawson would be likely to respond: Yes, sometimes but not at all always, and such stories may also be dangerous and lead people to fatal misunderstandings of their lives.

### Modified notions of narrativity

Philosopher Marya Schechtman’s reply to Strawson is an exemplary attempt to take an opponent’s position seriously, see what there is of value in it, and consequently adjust one’s own position in case this is judged to be needed. Schechtman welcomes several of the critical points that Strawson makes and uses them to refine her own theory. “Strawson points to many real deficiencies in existing narrative approaches”, she concedes ([7], p., 160).

Schechtman’s reasoning is intricate, detailed and hard to concisely capture. She distinguishes three key questions concerning the role of narrative in people’s lives: What a life-narrative is, what it means to *have* a life-narrative, and the implications of having, or failing to have, a life-narrative. In order to answer these questions she distinguishes between different “strengths” of life-narratives from weak, to middle-range to strong. Strawson would call the weak narrative “trivial” in so far as it mainly means that such a person “must be able to organize her life according to a fundamental implicit knowledge of the events in her history, or she will not be able to function well at even the most basic level” [p. 161]. The middle range position holds that a person “needs a certain understanding of how the events in her life hang together, an understanding that is mostly implicit but that she can access locally where appropriate….” [p. 161]. The strong view, finally, suggests that a person must consciously and actively undertake to live and understand her life as a story in the strong sense – with a unified theme and little or no extraneous material – if that life is to be meaningful." [p. 161].

While Strawson for different reasons would reject all three views, Schechtman attempts to defend a certain interpretation of the mid-range view, which she calls “the narrative self-constitution view”. In short, this sums up to the view that “we constitute ourselves as persons by forming a narrative self-conception according to which we experience and organize our lives” [p. 162]. But what, then, is a “narrative self-conception”? Schechtman is not very precise on this crucial point. “When I have a self-constituting narrative, what happens to me is interpreted not as an isolated incident, but as part of an ongoing story.” [p. 162] Schechtman links her view to the question of identity over time: “The implications of having a narrative, on this view, are that it provides the phenomenological unity of consciousness over time that constitutes personal survival and generates person-specific capacities such as moral agency, the ability to engage in prudential reasoning and in relations of compensation” [p. 167].

To sum up, Schechtman agrees with Strawson on several points, but defends a “refined” view of life-narrative as a story that unites events in our lives and has some degree of explanatory power concerning why we came to the point where we are, and where we will likely be in the future. She insists that having such a narrative is essential for leading a good life. Does this have any relevance for the discussion on narrativity and medicine? Now, this is where we will soon turn our attention.

Another attempt to hold on to an essential role for narrativity in our lives has recently been presented by Daniel D. Hutto. He labels his attempt a Narrative Self Shaping Hypothesis (NSSH), and it may be seen as a reply both to Strawson and to Schechtman. There is no need, in this context, to dwell on the subtleties of narrative theory, which may no doubt seem more sophisticated than relevant for our present analysis of the role of narrative in medicine. Interesting, however, is that Hutto seems to land close to Schechtman when he concludes that “.. there is no reason to give up on the idea that narrative capacities normally play an important role in enabling us to shape ourselves.” He continues:

A modest NSSH does not make any strong claims about the existence of narrative selves, or living out narratives in our lives or experiencing our lives narratively, and so on. It is quite possible to leave these ideas of Strong Narrativism to one side, while fully endorsing the view that a narrative understanding of reasons is an important basis for and natural means of certain forms of self-understanding and self-shaping [8].

## Narrative medicine

It is not surprising that the central concepts of the narrative turn – like narrative, narrativity, story, self-telling, meaning – were seen by a growing number of persons involved in analyzing and performing health care tasks as relevant also for the understanding of what goes on in clinical settings. The turn towards narrativity in philosophy and the social sciences not surprisingly coincided with the rise of medical ethics and a rapidly growing interest in “the patient as person”, which was also the title of Paul Ramsey’s book from 1970 [9]. The psychiatrist and anthropologist Arthur Kleinman, in his *The Illness Narratives* [10], argued convincingly for the need to pay close attention to the stories of ill persons. Philosopher and physician Eric Cassel’s *The Nature of Suffering and the Goals of Medicine* [11] was probably even more influential when he urged clinicians to search for a richer and more nuanced understanding of the overriding goal of medicine, human suffering. Neither of these was particularly occupied with narrativity as theory and had no ambition to claim a larger role for what was becoming called “narrative self-understanding”, or for that matter for “narrative skills”. They wrote in line with a broad tradition in what we now call medical humanities, with the purpose of restoring subjectivity in clinical medicine and increasing the interest of clinicians in what their patients have to say. They found themselves in no particular need of the tools of narratology or literary reception theory. Akin to these authors we find philosopher Stephen Toulmin who in a seminal essay from 1987, “Art and science in the practice of medicine”, eloquently argues that the practicing physician by necessity must rely on two epistemologies – that of the medically described body and that of the ill person’s inner world, his “biography”. If the physician is not interested in what the patient has to say, Toulmin concludes his essay, “then why be a physician at all?” [12].

One of the most influential contributions to the narrative turn in the social sciences, and in particular to the by then emerging field of medical humanities, is sociologist Arthur Frank’s *The Wounded Storyteller: Body, Illness, and Ethics,* which came in 1995. Frank wrote out of personal experience of serious illness. Illness is for Frank a threat to, in effect a wound in, the “body-self”, our bodily “being-in-the-world”. To heal that wound, to restitute bodily integrity, we need to formulate a story. These stories tend to take three major forms, of which the third, the quest story, is the preferred and the one that offers chances to become whole again. The quest story should be seen as an ideal type to which to aspire: “Quest stories meet suffering head on: They accept illness and attempts to *use* it. Illness is the occasion of a journey that becomes a quest” ([13], p., 115).

Frank emphasizes, over and over again, that the person forms herself through narratives. The outcome of disease will hence largely depend on what narrative the person “constructs”. Wounded story-tellers should ask themselves two questions: “What story do you wish to tell of yourself? How will you shape your illness, and yourself, in the stories you tell of it?” [p. 65]. Striking is how Frank emphasizes story-shaping as an act of will and the extent to which he regards this process also as a construction of personhood.

In 1998, British general practitioners Trisha Greenhalg and Brian Hurwitz published the anthology *Narrative Based Medicine: Dialogue and Discourse in Clinical Practice.* The essays cover a broad field of clinical practice, with an emphasis on doctor-patient interaction, as well as the training of medical students into the profession. Here, too, the back-ground is claimed to be the shortcomings of scientific medicine:

The relentless substitution during the course of medical training of skills that are fundamentally linguistic, empathic, and interpretive for those deeme" scientific", eminently measurable but unavoidably reductionist, should be seen as anything but a successful feature of the modern curriculum ([14], p 13).

Hence, when physician and literary scholar Rita Charon began her work in New York around the turn of the millennium in order to firmly anchor the idea of narrativity at the very center of clinical practice, the discourse on narrative and medicine was already well developed. Her critique of the real or alleged tendency in clinical practice to brush subjectivity aside is as eloquent as merciless:

Whether to protect themselves from the sadness of taking care of very sick people or to guarantee the objectivity of their clinical judgement, doctors seem to operate at a remove from sick and dying patients, divided from sick people by deep differences in how they conceptualize illness, what they think causes it, how they choose to treat it, and how they respond emotionally to its presence ([15], p., 6).

Rita Charon is, of course, fully aware of the beneficial aspects of modern medicine and in her clinical practice most probably takes these into use. She is right to point to the inherent risk, based on the ontology of scientific medicine, that the experiences - the “life worlds” - of those that medicine is there for become secondary to physical facts or even non-existent. Clinical practice, she is convinced, needs reform, needs a redressing of balance. This is the soil for narrative. It is launched as a remedy for a medicine that has forgotten how to approach its core task: the cure, prevention and alleviation of suffering. Only by way of an acquaintance with and skills to interpret narratives can medicine be reformed:

As doctors become more and more skilled in narrative capacities, they will improve their ability to develop accurate and comprehensive knowledge about patients, to reach patients, to become their trusted advocates, to navigate ethical uncertainty, and to be moved by all that they are privileged to do as doctors [16].

Obviously, the stakes are high here as well. I will propose that Charon’s initiative grew into a movement that is still with us. Much of value has come out of this attempt to emphasize the narrative aspects of medicine, but I will argue that there are good reasons to question some of the more far ranging ambitions of this reform project.

## Are the claims of narrative medicine justified?

We have concluded that the proponents of narrative medicine have ambitions to reform medical practice. In the essential contributions to narrative medicine, like the anthology *Stories Matter: The Role of Narrative in Medical Ethics* [16], and Rita Charon’s *Narrative Medicine: Honoring the Stories of Illness* [15] there can be identified three basic assumptions that seem essential to justify the claims of narrative medicine. They are the following:

1. Clinical practice has turned away from the experiences of ill persons, from the illness narratives, in favor of a reliance on scientific data and on a description of the medically constructed body in its normal and pathological functioning. If doctors lose interest in and contact with the lived realities of ill persons, clinical medicine will not be able to fulfill its basic goal of reducing suffering.
2. Human beings strive for an understanding of their lives and do it in a narrative way. Life-stories, narratives, that bind together events over time, and give continuity and provide meaning, are essential for a good life. Losing, or not managing to construct, such stories leads to suffering.

3. In order to restore the importance of human subjectivity in clinical practice, doctors as well as other health care professionals should take not only a keen interest in narratives, particularly literary narrative, but also be taught some of the tools of narrative analysis. Narrative competence is not reached only by reading but also by learning to go “deeper” into texts, how they are constructed and the way they exert their influence on the reader. The physician should be narratively skilled.

I will in the following basically support the first claim, though with a few reservations. I will argue that the third assumption is at best partially true, and that the second is probably false and perhaps also dangerous in its unqualified form, but less so if the claim is in line with the more modest proposals of Schechtman and Hutto.

### 1

Let us first examine what I have proposed as the first claim of narrative medicine, the alienation of doctors from their patients, their ethical insensitivity, and the need for stories, in particular literature, as a remedy to this.

Scientific medicine made its progress by ways of constructing a medicalized body, devoid of subjectivity, cut off from hope and longing and fear and hate [17, 18]. Since more than forty years, the risk that this entails has been widely acknowledged and addressed and programs have been launched to “humanize medicine”, starting with the ethics boom of the 70’s and 80’s, developing into the medical humanities movement of the early twenty-first century. Still, and in spite of continuing medical scientific progress, there is discontent and worries concerning the present state of clinical medicine and its direction during the coming years.

The critique is next to merciless. Arthur Frank declares: “I understand this obligation of seeking medical care as *a narrative surrender* and mark it as the central moment in modernist illness experience.” ([15], p., 6). Charon comes close to this interpretation when writing that “Many patients feel abandoned by their doctors, dismissed in their suffering, disbelieved when they describe their symptoms, or objectified by impersonal care.” [19] She repeatedly emphasizes this, and calls the doctor “… a person whose clinical training and clinical responsibility have spoiled his or her capacity to understand what living with sickness must be like” [19]. Already twenty years ago, Eric Cassell, in a sadly unacknowledged book called *The Place of Humanities in Medicine* [20], proposed that “The body has yielded its secrets in a consistent manner only since experimental and statistical methods were developed the *totally* divorce scientific generalizations from the individuality of persons” ([19], p., 17).

How valid, then, is the rather dark picture of medical practice that emerges in these quotations? It is beyond doubt that they reflect widely spread worries based on the rather gloomy chances for ill persons to experience that their physicians show an interest in and respect for their unique experience, as well as many doctors´ lack of attempts to understand the alienation from ordinary life that often comes with serious illness. Physicians seem all too often to focus exclusively on disease, and brush illness aside.

But even when admitting this as a risk, many clinicians would object and argue that they certainly are interested in what their patients have to say, but that time is almost always scarce, and that their patients primarily want them to repair what is wrong in their bodies, thereby permitting a restoration of the kind of life they had before falling ill. In Sweden for example, repeated surveys show a high degree of patient satisfaction with their clinical encounters. Such circumstances as a shortage of time for the consultation and often a lack of continuity do not seem to change this.

It must also be taken into account that the variations in medical practice are huge, not only between different clinicians but between different disciplines as well. In the primary care consultation, technology is usually not dominant, and the mutual dialogue is at the center, even if time often is too short also for the family physician [21, 22]. This does not mean that Charon’s urge that doctors explore the ill person’s experience is not well founded, rather that it is difficult to make sweeping general conclusions concerning physicians’ willingness and ability to engage with their patients in a more mutual way.

We may conclude that even though the critique of contemporary clinical practice often lacks nuances and ignores the disciplinary and personal differences in how medicine is practiced and what ill persons want from their doctors, it remains clear that a redressing of epistemological balance is needed in order for medicine to cure, alleviate and console – and in doing so, respect the unique value of each suffering human being.

### 2

Should we accept the heavy critique that Galen Strawson and Angela Woods, as well as others, have launched against some of the claims of the narrativity movement? And if we do, what would the consequences be for the agenda of narrative medicine?

I believe that the former question ought to get an affirmative reply, with some reservations. Both Strawson and Woods have identified weaknesses in the ambitions of “the narrative turn”, and these weaknesses have consequences also for narrative medicine. Charon flatly states that: “Telling our story does not merely document who we are; it helps to make us who we are” ([16], p., 69), and also: “All who want to learn of the self may be deeply interested in the unique, genuine strand of life-telling that goes on in medicine.” ([16], p., 78) Reading Charon, it becomes obvious how deeply narrative medicine has been influenced, even permeated, by the assumptions of Bruner, Frank, Taylor and other theorists of narrativity. For them, the reason why narratives are so important for our understanding of others is that we are all in a qualified way narrative. Both the Descriptive and Ethical narrativity theses are, implicitly or explicitly, present in the writings of the proponents of narrative medicine.

But all humans are not narratively inclined in the sense that is taken for granted, and in case they are, it seems highly dubious whether this is always a good thing. If we accept Strawson’s objection, it means that a narrative inclination - in his words: being a Diachronic - might just as well tempt someone to self-deception, into a mis-representation of his or her life.

However, we have seen that, for example, Schechtman and Hutto are able to rescue a weaker interpretation of the narrativity thesis. Human beings do to some extent “self-constitute” (Schechtman) or “self-shape”(Hutto) themselves by means of narrative. Strawson reminds us that people are fundamentally different in this respect, and doctors must take this into consideration. Some of the claims of narrative medicine must be regarded skeptically, exactly for this reason. But to deny that stories matter to some degree for all humans, and particularly for those who have fallen ill, is to deny the obvious.

Therefore, it is the leap from suggesting the value of doctors listening to their patients’ stories, which seems next to trivially true by any reasonable definition of “story”, to claiming that all or most patients by necessity are “narrative” and need to construct “life-stories” that is deeply problematic. At best, it increases the interest in what ill persons have to say and not much more; at worst, it will tend to press patterns of interpretation on persons who neither want nor should be thought of as Narratives, in Strawson’s sense.

### 3

Rita Charon is clear about the objectives of narrative medicine. Those who practice narrative medicine need to possess narrative tools. These are variably called “narrative competence”, “narrative skills” and “narrative acts”. Charon admits that clinicians who are already over-burdened by work and by the large amount of medical knowledge needed to keep continuously updated could not be expected to study narratology in depth. But she insists: “We want to make them transparent to themselves as readers, and we want to equip them with the skills to open up the stories of their patients to nuanced understanding and appreciation” ([16], p., 110). The precise meaning of such “skills” remains obscure. The familiarity with texts and some basic knowledge of how these may be constructed, a sensitivity to language, some training in writing literary texts oneself - this may of course be called “narrative skills”. But one not seldom gets the impression that more qualified tools are needed, some of the tools of narratology, which only literary theorists are in possession of.

There is, according to Charon, no tension between these narrative skills and an interest in the story, the ethical dimensions and the possible associations that can appear. It seems as if Charon looks upon the tools of narrative theory as an equivalent of, in fact a part of, what physicians need to acquire by clinical training. Without these two, the clinician does not get much out of the encounter, with patient or with text. The impression is that of literary texts as closed treasures that one would need the training of literary scholars to open up and benefit from. If this were the case, it would, for example, by definition be more valuable for a group of medical students reading literature as part of their curriculum to have a literary theorist as supervisor as compared to some other literarily experienced and deeply interested person, perhaps a clinician. I find this highly contestable and Charon doesn’t present any convincing arguments for her position on this point.

Could empirical evidence support Charon’s claims? The problem is that empirical studies in this area are almost unavoidably biased, confounded and unreliable. An instructive case is a study made on fourth year medical students, and published under the title “Narrative medicine as a means of training medical students toward residency competencies” [23]. Noteworthy is that the number of students participating was twelve, the course was given during a month and was an elective, and that half of the students had completed undergraduate studies in the humanistic field. Hence, the group was highly unrepresentative and of course, as their choice showed, unusually motivated. These students were taught by “six faculty members trained in narrative medicine”, by reading literary texts of different sorts, writing and discussing. It is unclear to what extent specific narratological knowledge was included and which was the competence of the teachers in narrative skills. The students were then evaluated through surveys and focus group discussion at the end of the course, and some questions were renewed one and a half year later. Not surprisingly the students thought that they had improved “communication skills” as well as their capacity to emphasize, be “patient-centered” and that they had has “developed their personality”.

Such results are of course to be expected from a highly selected group, which got so much attention. No conclusions whatsoever can be drawn from them, and even if some of the most obvious shortcomings of this study may be overcome, the obstacle to any solid knowledge about outcomes of reading are insurmountable. The enthusiasm with which the results are presented are illuminating for the lack of skepticism that characterizes the narrative medicine movement. Of course, nothing excludes that the results really are very beneficial and that narrative training, in which ever way it is done, is superior to all other ways of increasing clinical skills and judgement among medical students. But a lower tone should certainly be motivated, as proof is totally lacking.

There are, I believe, some sound reasons why scholars like Wayne Booth and Louise Rosenblatt, as well as Anders Pettersson [24]. and Frank Palmer [25], do not assume that the beneficial effects of reading literary texts, if there are such, necessarily depend on narrative skills in any more qualified sense. Their focus is on the plot, the presentation to the reader of possible worlds where fictive persons act in ways that evoke our curiosity and demand our understanding. My suspicion is that they would even be suspicious of a reading that is too eager to employ narrative tools. In contrast to what Charon takes for granted, the narratological theory apparatus may at times stand in the way for the plot. Of course, this does not exclude the value of some degree of interest in how texts are constructed, and of course not of a wide literary experience, but it seems both pretentious and misleading to call such a basic familiarity “narrative competence”.

At the end we must ask ourselves if we have good reasons to believe that reading literature will increase physicians´ ability to understand and respond to illness and suffering? In 2000, Jemeljan Hakemulder published a summary of the results of a number of studies on the effects of reading, *The Moral Laboratory.* Hakemulder selected a large number of reading experiments, which were conducted in order to find out what effects reading had on the ethical capacity of the readers. There is a lot to be said about the chances of such studies to show anything at all with a reasonable amount of certainty, given the large number of confounding factors that plague them. A major weakness of his study is that he neither investigated what reading during a longer period of time may mean, nor did he look at the effects of reading in groups with supervisors. This limits the value of his study, but the result – small or no beneficial effects - still raises important questions for those who take the beneficial effects of reading for granted [26].

Another skeptic, Suzanne Keen, in her extensive investigation *Empathy and the Novel,* laconically writes that.

While I certainly do not think that novels have a primarily negative influence on readers, I observe that their impact is considerably more unruly than advocates of narrative ethics would lead us to believe. ([27], p. 68).

To summarize, the proposal that literary experience is beneficial for physicians is too sweeping. We have scarce evidence that ethical reasoning or empathic capacity is in general affected in a positive way, though it may be under certain carefully specified circumstances. A number of questions, concerning what, when and how to read, must be answered. That there is a *potential* for literature to stimulate interest in ill persons’ stories seems clear [18]. Such an interest is undoubtedly of value for clinicians. It is hard to contest that physicians who are able to listen attentively and who show interest in and respect for what their patients tell them perform better than those who do not. That specific narrative skills, like possessing the tools of narrative theory, should be either a necessary or sufficient, or even contributing, condition for this interest and knowledge about human beings to arise from reading and writing seems highly unlikely. Attempts to show this are almost exclusively based on self-report and run into massive methodological difficulties.

## Some conclusions

Intellectual fashions come and go. What I have here called the narrative turn has many of the characteristics of such a fashion. It quickly attracted many researchers, the concept spilled over its limits and became a unifying key word to signal belonging and depth of thought, while at the same time being more and more diluted. It should, however, be remembered that narrative research is older than this new interest, that a lot of narrative research has avoided the pitfalls that characterize intellectual fashions and that a generally increased interest in the concept has in some ways been very beneficial. The wave of interest in narrativity has also provoked a counter-reaction that has in some ways been of more interest than the original movement. I have paid a lot of attention to Strawson’s article from 2004, for the reason that I regard it as an example of a strongly polemic but also fertile reaction to an academic hegemony. The examples of responses to Strawson that are here mentioned, Marya Schechtman and Daniel Hutto, convincingly show us that intellectual exchange can lead to increased conceptual clarity and hence improved understanding.

For clinical medicine, an obvious advantage has been that the interest in narrativity has paved the way for, as well as being a result of, an increasing interest in human subjectivity. As I have shown, there is a need to redress an epistemological unbalance in practical medicine, and to this end patient stories are of crucial importance. Bloggs on the internet overflow with such stories, which may be of value to all who deal with health and disease, apart from being of great general human interest. Also, being in the company of good fiction has all the potentials of improving clinical practice, however hard it will be to prove this in a solid empirical way. Medical humanities have come to stay and literary fiction is a central component of this field.

However, narratological skills in any more qualified sense are no necessary requirement for gaining such practically valuable knowledge. It is “the company we keep” (Wayne Booth), the invitation to be emotionally moved while at the same time retaining the distance that fiction includes – these are the key elements that make literary narrative valuable for clinicians. The ambitions of narrative medicine deserve our support, but the movement would do well with a bit more of critical skepticism concerning some of its most cherished assumptions.

## Data Availability

All data generated or analysed in this study are included in this published article.
